# Study of the *in Vitro* Antiplasmodial, Antileishmanial and Antitrypanosomal Activities of Medicinal Plants from Saudi Arabia

**DOI:** 10.3390/molecules171011379

**Published:** 2012-09-25

**Authors:** Nawal M. Al-Musayeib, Ramzi A. Mothana, Shaza Al-Massarani, An Matheeussen, Paul Cos, Louis Maes

**Affiliations:** 1 Department of Pharmacognosy, College of Pharmacy, King Saud University, P.O. Box 2457, Riyadh 11451, Saudi Arabia; Email: nalmusayeib@ksu.edu.sa (N.M.A.-M.); salmassarani@ksu.edu.sa (S.A.-M.); 2 Department of Pharmacognosy, Faculty of Pharmacy, Sana’a University, P.O. Box 33039, Sana’a, Yemen; 3 Laboratory for Microbiology, Parasitology and Hygiene (LMPH), Faculty of Pharmaceutical, Biomedical and Veterinary Sciences, Antwerp University, Universiteitsplein 1, 2610 Wilrijk-Antwerp, Belgium; Email: an.matheeussen@ua.ac.be (P.C.); paul.cos@ua.ac.be; louis.maes@ua.ac.be (L.M.)

**Keywords:** medicinal plants, antiprotozoal, antiplasmodial, antileishmanial, antitrypanosomal, crude extracts, Saudi Arabia

## Abstract

The present study investigated the *in vitro* antiprotozoal activity of sixteen selected medicinal plants. Plant materials were extracted with methanol and screened *in vitro* against erythrocytic schizonts of *Plasmodium falciparum*, intracellular amastigotes of *Leishmania infantum* and *Trypanosoma cruzi* and free trypomastigotes of *T. brucei*. Cytotoxic activity was determined against MRC-5 cells to assess selectivity*.* The criterion for activity was an IC_50_ < 10 µg/mL (<5 µg/mL for *T. brucei*) and a selectivity index of ≥4. Antiplasmodial activity was found in theextracts of *Prosopis juliflora* and *Punica granatum*. Antileishmanial activity against *L. infantum* was demonstrated in *Caralluma sinaica* and *Periploca aphylla.* Amastigotes of *T. cruzi* were affected by the methanol extract of *Albizia lebbeck* pericarp, *Caralluma sinaica*, *Periploca aphylla* and *Prosopius juliflora*. Activity against *T. brucei* was obtained in *Prosopis juliflora*. Cytotoxicity (MRC-5 IC_50_ < 10 µg/mL) and hence non-specific activities were observed for *Conocarpus lancifolius*.

## 1. Introduction

Protozoal infections are a worldwide health problem, particularly in the Third World countries [1,2,3,4], which account for approximately 14% of the world population whom are at risk of infection. Therefore, a great concern has been expressed by the WHO, as they are considered neglected tropical diseases [5]. These tropical diseases may impose negative impacts on the socioeconomic status of infected people or those who are subject to infection [6]. These diseases, for example, may affect the quality of people’s life and their development in developing countries. Various studies have been conducted on protozoal diseases, including leishmaniasis, malaria, chagas and sleeping sickness. These diseases are considered as major killing factors, particularly in view of the fact that various difficulties are associated with controlling the sources of infection, the high cost of treatment/prevention and poor compliance. In addition, other difficulties include drug resistance, low efficacy and poor safety, which may retard treatment [7]. Therefore, there is always need for the development of new and more effective drugs [8]. In this respect, natural products offer good sources for new drug discovery. Various antiparasitic drugs have been developed from natural sources, including quinine, artemisinin and atovaquone as antimalarials and amphotericin B as antileishmanial drug. It is estimated that approximately 60% of the world population still use traditional remedy methods, mainly medicinal plants or their products, as they cannot afford to buy pharmaceutical products [9,10]. This study focuses on an *in vitro* investigation of the antiprotozoal activity of 16 medicinal plants.

## 2. Results and Discussion

### 2.1. Results

In continuation of our previous study on antiprotozoal activity of medicinal plants [11], sixteen plant species that are used in traditional medicine were selected for the current study (Table 1). Crude methanol extracts of the selected plants were evaluated in an integrated *in vitro* screen for antileishmanial, antiplasmodial and antitrypanosomal potential (Table 2). Extracts of *A. lebbeck* pericarp, *C. sinaica*, *P. aphylla*, *P. juliflora* and *P. granatum* exhibited antiprotozoal activities in one or more models (Table 2).

#### 2.1.1. Antimalarial Activity

In this study, the crude methanol extracts of *P. juliflora* and *P. granatum*, exhibited the greatest activity against *P. falciparum* and showed high selectivity index (IC_50_ 4.1, 6.7 µg/mL, SI 12.2, >9.6 respectively).

**Table 1:** List of plants screened and their traditional uses.

Plant species	Voucher specimen	Family	Part screened	Medicinal uses
*Albizia lebbeck* (L.) Benth.	15101	Leguminosae	T, S	As astringent, and for pile, diarrhea, dysentery, gonorrhea, spongy and ulcerative gums, and night blindness ^a^
*Cadaba farinosa* Forssk.	15111	Capparaceae	L, S	As stimulant, purgative, anthelmintic, antisiphilitic, antirheumatic emmenagogue and aperients, and for anthrax, cough, fever and dysentery ^a^
*Cadaba glandulosa* Forssk.	15102	Capparaceae	L, S	As anthelmintic ^b^
*Caralluma sinaica* (Decne.)	15130	Asclepiadaceae	L	As hypoglycemic ^c^
*Celtis africana* N.L.Burm.	15144	Cannabaceae	L, S	For rheumatism, cancer and toothache ^d,e,f^
*Conocarpus lancifolius* Engl.	15103	Combretaceae	T	Unknown
*Cordia sinensis* Lam.	15104	Boraginaceae	L, S	For rheumatism, painful menstruation, bladder diseases, gastric ulcers and malaria ^g,h^
*Iris germanica* L.	15161	Iridaceae	R	For treatment of cancer, inflammation, bacterial and viral infections ^i^
*Nigella sativa* L.	15132	Ranunculaceae	Se	As digestive, stimulant, carminative, aromatic, diuretic, diaphoretic, stomachic, anthelmintic, as circulatory and immune system support, analgesic, anti-inflammatory, anti-allergic, antioxidants, anticancer and antiviral ^a,j^
*Periploca aphylla* Decne.	15166	Asclepiadaceae	L, S	As stomachic, purgative and for cerebral fever ^a^
*Phoenix dactylifera* L.	15172	Arecaceae	Se	For infectious diseases, atherosclerosis, diabetes, hypertension and cancer and, as tonic aphrodisiac, and purgative ^k,l^
*Prosopis juliflora* (Sw.) DC.	15110	Leguminosae	T	For eye problems, open wounds, dermatological ailments, and digestive problems ^m^
*Punica granatum* L.	15156	Punicaceae	T	acidosis, dysentery, microbial infections, diarrhoea, helminthiasis, haemorrhage, and respiratory pathologies ^n^
*Ribes nigrum* L.	15137	Grossulariaceae	T	For throat inflammation and repiratory tract ailment ^q^
*Salvadora persica* Wall.	15112	Salvadoraceae	L, S	As aromatic, deobstruent, carminative, diuretic, anthelmintic and anti-inflammatory and for tumors and renal stones ^a,b^
*Zingiber officinale* Roscoe	15178	Zingiberaceae	R	As anti-emetic, stomachic, carminative ^r^

L: Leaves; R: Roots or rhizomes; S: Stems; Se: Seeds; T: Fruits. ^a^ Mossa *et al*., (1987) [12]; ^b^ Al-Yahya *et al*., (1990) [13]; ^c^ Habibuddin *et al*., (2008) [14]; ^d^ Arnold *et al*., (1984) [15]; ^e^ Srinivas *et al*.; (2007) [16]; ^f^ Yineger *et al*., (2008)[17]; ^g^ Do Vale *et al*., (2012) [18]; ^h^ Orwa, *et al*., (2009) [19]; ^i^ Ibrahim, *et al*., (2012) [20]; ^j^ Bakhotmah *et al.*, (2010) [21]; ^k^ Al-Qarawi *et al*., (2004) [22]; ^l^ Ardekani *et al*., (2010) [23]; ^m^ M. Sathiya *et al*., (2011) [24]; ^n^ Sánchez-Lamar *et al.*, (2008) [25], ^q^ Šarić-Kundalić *et al.*, (2011) [26]; ^r^ A.M. Ageel, *et al*., (1987) [27].

**Table 2:** Antiprotozoal activity of the extracts of the investigated plants and their cytotoxicity against MRC-5 cell lines.

Plant species	*P. falciparum*		*L. infantum*		*T. cruzi*		*T. brucei*		MRC-5
	IC_50_	SI	IC_50_	SI	IC_50_ (µg/mL)	SI	IC_50_	SI	IC_50 _
*Albizia lebbeck*	37.9 ± 4.3	-	50.8 ± 7.3	-	8.7 ± 1.1	3.7	8.1 ± 2.3	4.0	32.0 ± 3.5
*Cadaba farinose*	31.4 ± 2.5	1.1	>64.0	-	28.6 ± 4.1	1.2	10.6 ± 1.6	3.1	32.9 ± 4.2
*Cadaba glandulosa*	61.5 ± 5.6	>1.0	>64.0	>1	36.5 ± 3.6	>1.8	16.4 ± 2.0	>3.9	>64.0
*Caralluma sinaica*	>64.0	-	8.1 ± 2.1	2.5	7.3 ± 1.7	2.8	7.7 ± 1.2	2.7	20.5 ± 2.3
*Celtis Africana*	29.9 ± 5.2	>2.1	>64.0	>1	29.4 ± 5.4	>2.2	>64.0	>1	>64.0
*Conocarpus lancifolius*	10.3 ± 1.9	-	>64.0	-	32.2 ± 3.1	-	35.2 ± 5.3	-	7.2 ± 1.1
*Cordia sinensis*	>64.0	>1	>64.0	>1	33.9 ± 2.6	>1.9	32.0 ± 4.7	> 2.0	>64.0
*Iris germanica*	46.6 ± 4.1	>1.4	32.2 ± 6.7	>2	24.6 ± 1.7	>2.6	8.2 ± 1.2	>7.8	>64.0
*Nigella Sativa*	>64.0	>1	>64.0	>1	>64.0	>1	>64.0	>1	>64.0
*Periploca aphylla*	22.6 ± 2.3	1.1	6.0 ± 1.5	4.0	8.1 ± 1.9	3.0	7.1 ± 2.3	3.4	23.9 ± 3.4
*Phoenix dactylifera*	>64.00	>1	32.5 ± 4.3	>2.0	46.5 ± 7.3	>1.4	36.2 ± 4.7	>1.8	>64.0
*Prosopis juliflora*	4.1 ± 0.9	12.2	35.3 ± 2.6	1.4	10.4 ± 1.3	4.8	2.0 ± 0.4	24.9	49.8 ± 6.2
*Punica granatum*	6.7 ± 1.7	>9.6	> 64.0	>1	35.2 ± 4.8	>1.8	34.3 ± 7.2	>1.9	>64.0
*Ribes nigrum*	>64.0	>1	>64.0	>1	>64.0	>1	>64.0	>1	>64.0
*Salvadora persica*	56.4 ± 7.9	1.1	> 64.0	>1	30.1 ± 8.2	2.13	32.0 ± 5.9	>2.0	>64.0
*Zingiber officinale*	> 64.0	-	> 64.0	-	>64.0	-	39.4 ± 6.3	-	34.3 ± 5.7
Chloroquine	0.3 ± 0.1		-		-		-		-
Miltefosine	-		3.3 ± 0.7		-		-		-
Benznidazole	-		-		2.2 ± 0.5		-		-
Suramin	-		-		-		0.03 ± 0.02		-
Tamoxifen	-		-						11.0 ± 2.3

IC_50_ values of reference drugs are expressed in µM/mLconcentrations.

#### 2.1.2. Antileishmanial Activity

The methanol extract of *P. aphylla* showed the greatest activity against *L. infantum*(IC_50_ 6.0 µg/mL, SI 4.0). In addition, *C. sinaica* extract gave IC_50_ 8.1 and low selectivity index (SI 2.6).

#### 2.1.3. Antitrypanosomal Activity

The results of this study demonstrated that *T. b. brucei* is more sensitive than *T. cruzi* towards the extract of *P. juliflora*, which showed pronounced activity against *T. cruzi* with high selectivity (IC_50_ 2.0 µg/mL, SI 25.0) and moderate activity against *T. cruzi* with IC_50_ of 10.4 µg/mL and lower selectivity (SI 4.8). Furthermore, *T. cruzi* was sensitive towards the methanol extracts of *A. lebbeck* pericarp, *C. sinaica* and *P. aphylla* (IC_50_ 8.7, 7.3 and 8.1 µg/mL, respectively) and showed good selectivity (SI ca. 4). Meanwhile, marginal activity was exhibited by, *A. lebbeck* pericarp, *Cadaba farinose*, *C. sinaica*, *Iris germanica* and *P. aphylla*,with IC_50_ values between 10.6 and 7.1 μg/mL and SI values between 3.0 and >7.8 (Table 2).

#### 2.1.4. Cytotoxicity of Plant Extracts

The methanol extract of *C. lancifolius* demonstrated a noticeable cytotoxic effect against MRC-5 cells (IC_50_ of 7.2 µg/mL).

### 2.2. Discussion

Many medicinal plants display effective pharmacological potential for the treatment of different diseases caused by protozoan parasites [28,29]. Therefore, results of numerous global studies on the effect of various medicinal plants that exhibit antiprotozoal activity have been reported [30,31,32,33,34,35]. In addition, the Arabian Peninsula has a rich flora of different medicinal plants, including plants with antiprotozoal potential. Therefore, this research has focused on screening 16 of these plants for their antiplasmodial, antileishmanial and antitrypanosomal activities, as part of our continued research in this area [28]. Results reported here have shown that the plant extracts displayed different levels of antiprotozoal activity. It is important to mention that to the best of our knowledge, this study represents the first report on antiprotozoal activities for most part of the investigated plants. Although few plants are partly investigated, existing knowledge is in many cases very limited. Based on the activity (IC_50_) and selectivity, five plant extracts could be considered as promising and interesting to be further elaborated through purification and biological evaluation on an individual compound basis.

There is no reported evidence of antiprotozoal activity of *P. juliflora* phytochemicals. The methanol extract of the *P. juliflora*, collected from Saudi Arabia, exhibited the greatest antiplasmodial activity with the highest selectivity index (IC_50_ 4.1 μg/mL, SI 12.2). Our result is in agreement with data reported recently by Ramazani *et al.* [36], which showed significant antiplasmodial activity for a hydro-alcoholic extract of this species collected from Khouzestan in Iran, against both chloroquine-resistant (K1) and chloroquine-sensitive *P. falciparum* (CY27) (IC_50_ 14.78 and 4.68 μg/mL, respectively). Additionally, this is the first report on the antitrypanosomal activity of *P. juliflora* extract against both *T. cruzi* and *T. brucei*. It showed the highest selectivity index with pronounced activity against *T. brucei* (IC_50_ 2.0 μg/mL, IS 24.9). In a large number of plants with antiprotozoal activity, the therapeutic value is due to the presence of alkaloids. Juliflorine and its isomer julifloricine are the main alkaloids of *P. juliflora* [37], making them obvious targets for a compound-based follow-up.

Other interesting source of antiplasmodial activity is *P. granatum* fruit rind (IC_50_ 6.7 μg/mL, IS > 9.6). In India it is used as anti-malarial home remedy and is reported to successfully control *P. falciparum* and *P. vivax* infections [38,39]. Dell’Agli *et al.* attributed this anti-malarial effect to the anti-parasitic activity and an inhibitory action on the pro-inflammatory mechanisms involved in the onset of cerebral malaria [40]. Ellagitannins and in particular ellagic acid, and punicalagin greatly contribute to the antimalarial effect of *P. granatum* fruits rind [40,41,42]. Our result is in agreement with reports on its antiplasmodial effect. However, though tannins such as ellagic acid were reported [43] to exhibit pronounced antileishmanial activity against intracellular amastigotes of *L. donovani*, in our study, the methanol extract of *P. granatum* fruits rind was devoid of such effect on *L. infantum* amastigotes.

Our antiparasitic screening revealed remarkable *in vitro* antileishmanial and anti-*T. cruzi* activity for *P. aphylla*,but with a moderate selectivity index (IC_50_ 6.0, 8.0 µg/mL SI 4.0, 3.0, respectively) (Table 2). These findings are in agreement with literature data for other *Periploca* species published recently. Abdel-Sattar *et al.* [44] reported antitrypanosomal activity of the methanol extract of *P. somaliensis* growing in Saudi Arabia against *T. brucei*, with IC_50_ 7.1 µg/mL and SI value of 6.4. Additionally, antileishmanic effects of *P. graeca* against *L. major* were reported by Demiric *et al*. in 1998 [45], while no reported data is found on its activity against *T. cruzi* and *L. infantum*. Phytochemical investigation of *Periploca* species has shown that this genus mainly contains cardenolides and pregnane glycosides [46,47]. On the other hand, the crude extract of *C. sinaica* showed good antileishmanial effect and antitrypanosomal activity against both trypanosome species albeit with low selectivity (SI < 3), while, *P. falciparum* was insensitive to this extract. Literature data reported by Abdel-Sattar in 2008 [48] on another species of *Caralluma*, namely *C.* tuberculata, revealed no antiplasmodial and antitrypanosomal activity of its methanol extract and it showed high toxicity on MRC5 (IC_50_ 0.8 μg/mL), even though Abdel-Sattar *et al.* reported moderate antitrypanosomal activity of the CHCl_3_ soluble fraction of *C.* tuberculata (IC_50_ 3.5 μg/mL, SI of 17.9), and isolated acylated pregnane glycosides that showed weak antiparasitic activity [48]. Additionally, Abdel-Sattar *et al.* reported a pronounced antitrypanosomal effect of the pregnane glycosides penicilloside E, isolated from *C. penicillata*, and caratuberside C, from *C. tuberculata* (IC_50_ 1.01, 1.85 μg g/ mL, respectively) [49]. The literature reports *in vitro* antiplasmodial activity for a number of *Albizia* species, including *A. gummifera* and *A. saman* [50,51,52]. The antiplasmodial activity of *Albizia* species is attributed to spermine alkaloids including budmunchiamine K and its derivatives 6-hydroxybudmunchiamine K, 5-normethylbudmunchiamine K, 6-hydroxy-5-normethylbudmunchiamine K and 9-normethylbudmunchiamine [51,52]. In the current study, however, methanolic extracts of *A.* *lebbeck* seeds and pericarp were found to have no antiplasmodial activity. On the other hand, *A.* *lebbeck* pericarp extract showed antitrypanosomal activity, which was in agreement with the results obtained by Freiburghaus *et al*. [53], who reported promising effect of the lipophilic extracts of *A. gummifera*.

## 3. Experimental

### 3.1. Plant Materials

Fifteen plants (Table 1) were selected and collected randomly from different areas of Saudi Arabia in March and June 2008. In addition, dried *Zingiber officinale* root was obtained from a local market. Plant materials were identified by a taxonomist at the Pharmacognosy Department, Colleges of Pharmacy, King Saud University, Saudi Arabia. Voucher specimens were deposited at department. Table 1 shows the botanical name, voucher specimen, plant part screened and the reported medicinal uses of the plants.

### 3.2. Extraction of Plant Materials

The air-dried, powdered plant material (100 g) was placed in a Soxhlet apparatus and extracted with refluxing methanol (500 mL) for 8 h. The methanolic extract was then filtered off and concentrated using rotatory evaporator and freeze dried to remove any traces of methanol. The dried extracts were stored at −20 °C until used. Stock solutions for screening were prepared in DMSO at concentration 20 mg/mL.

### 3.3. Reference Drugs

For the different tests, appropriate reference drugs were used as positive control: tamoxifen for MRC-5, chloroquine for *P. falciparum*, miltefosine for *L. infantum*, benznidazole for *T. cruzi* and suramin for *T. b. brucei*. All reference drugs were either obtained from the fine chemical supplier Sigma or from WHO-TDR.

### 3.4. Biological Assays

The integrated panel of microbial screens and standard screening methodologies were adopted as previously described [38]. All assays were performed in triplicate, at the Laboratory of Microbiology, Parasitology and Hygiene at the University of Antwerp, Belgium. Plant extracts were tested at 5 concentrations (64, 16, 4, 1 and 0.25 µg/ mL) to establish a full dose-titration and determination of the IC_50_ (inhibitory concentration 50%). The concentration of DMSO did not exceed 0.5%. The selectivity antiprotozoal potential was assessed by simultaneous evaluation of cytotoxicity on a fibroblast (MRC-5) cell line. The criterion for activity was an IC_50_ < 10 µg/mL (<5 µg/mL for *T. brucei*) and a selectivity index of ≥4.

### 3.5. Antileishmanial Activity

*L.infantum* MHOM/MA(BE)/67 amastigotes were collected from the spleen of an infected donor hamster and used to infect primary peritoneal mouse macrophages. To determine *in vitro* antileishmanial activity, 3 × 10^4^ macrophages were seeded in each well of a 96-well plate. After 2 days outgrowth, 5 × 10^5^ amastigotes/well, were added and incubated for 2 h at 37 °C. Pre-diluted plant extracts were subsequently added and the plates were further incubated for 5 days at 37 °C and 5% CO_2_. Parasite burdens (mean number of amastigotes/macrophage) were microscopically assessed after Giemsa staining, and expressed as a percentage of the blank controls without plant extract.

### 3.6. Antiplasmodial Activity

Chloroquine-resistant *P. falciparum* 2/K 1-strain was cultured in human erythrocytes O^+^ at 37 °C under a low oxygen atmosphere (3% O_2_, 4% CO_2_, and 93% N_2_) in RPMI-1640, supplemented with 10% human serum. Infected human red blood cells (200 µL, 1% parasitaemia, 2% haematocrit) were added to each well and incubated for 72 h. After incubation, test plates were frozen at −20 °C. Parasite multiplication was measured by the Malstat method [54,55].

### 3.7. Antitrypanosomal Activity

*Trypanosoma brucei* Squib-427 strain (suramin-sensitive) was cultured at 37 °C and 5% CO_2_ in Hirumi-9 medium [56], supplemented with 10% fetal calf serum (FCS). About 1.5 × 10^4^ trypomastigotes/well were added to each well and parasite growth was assessed after 72 h at 37 °C by adding resazurin [57]. For Chagas disease, *T. cruzi* Tulahuen CL2 (benznidazole-sensitive) was maintained on MRC-5 cells in minimal essential medium (MEM) supplemented with 20 mM L-glutamine, 16.5 mM sodium hydrogen carbonate and 5% FCS. In the assay, 4 × 10^3^ MRC-5 cells and 4 × 10^4^ parasites were added to each well and after incubation at 37 °C for 7 days, parasite growth was assessed by adding the β-galactosidase substrate chlorophenol red β-D-galactopyranoside [58]. The color reaction was read at 540 nm after 4 h and absorbance values were expressed as a percentage of the blank controls.

### 3.8. Cytotoxicity Assay

MRC-5 SV2 cells were cultivated in MEM, supplemented with L-glutamine (20 mM), 16.5 mM sodium hydrogen carbonate and 5% FCS. For the assay, 10^4^ MRC-5 cells/well were seeded onto the test plates containing the pre-diluted sample and incubated at 37 °C and 5% CO_2_ for 72 h. Cell viability was assessed fluorimetrically after 4 h of addition of resazurin. Fluorescence was measured (excitation 550 nm, emission 590 nm) and the results were expressed as % reduction in cell viability compared to control.

## 4. Conclusions

In conclusion, the current work has led to the identification of five plant species/parts exhibiting relevant antiprotozoal activity, namely *A. lebbeck* pericarp, *C. sinaica*, *P. aphylla*, *P. juliflora* and *P. granatum.* Our results further support the idea that medicinal plants can be promising sources of potential antiplasmodial antileishmanial and antitrypanosomal agents. The present results will form the basis for selection of plant species for further investigation in the potential discovery of new natural bioactive compounds.
